# Measuring teamwork for training in healthcare using eye tracking and pose estimation

**DOI:** 10.3389/fpsyg.2023.1169940

**Published:** 2023-05-31

**Authors:** Kerrin Elisabeth Weiss, Michaela Kolbe, Quentin Lohmeyer, Mirko Meboldt

**Affiliations:** ^1^Product Development Group Zurich, ETH Zurich, Zurich, Switzerland; ^2^Simulation Center, University Hospital Zurich, Zurich, Switzerland

**Keywords:** teamwork, training, eye tracking, pose estimation, simulation, feedback, technology, behavior measurement

## Abstract

Teamwork is critical for safe patient care. Healthcare teams typically train teamwork in simulated clinical situations, which require the ability to measure teamwork via behavior observation. However, the required observations are prone to human biases and include significant cognitive load even for trained instructors. In this observational study we explored how eye tracking and pose estimation as two minimal invasive video-based technologies may measure teamwork during simulation-based teamwork training in healthcare. Mobile eye tracking, measuring where participants look, and multi-person pose estimation, measuring 3D human body and joint position, were used to record 64 third-year medical students who completed a simulated handover case in teams of four. On one hand, we processed the recorded data into the eye contact metric, based on eye tracking and relevant for situational awareness and communication patterns. On the other hand, the distance to patient metric was processed, based on multi-person pose estimation and relevant for team positioning and coordination. After successful data recording, we successfully processed the raw videos to specific teamwork metrics. The average eye contact time was 6.46 s [min 0 s – max 28.01 s], while the average distance to the patient resulted in 1.01 m [min 0.32 m – max 1.6 m]. Both metrics varied significantly between teams and simulated roles of participants (*p* < 0.001). With the objective, continuous, and reliable metrics we created visualizations illustrating the teams’ interactions. Future research is necessary to generalize our findings and how they may complement existing methods, support instructors, and contribute to the quality of teamwork training in healthcare.

## 1. Introduction

Teamwork is critical for safe patient care. Professionals from different “tribes” must team up — oftentimes on the spot — and work together to achieve excellent patient care (Rosen et al., 2018). Poor teamwork is a considerable risk for patient safety; great teamwork is an enormous asset, particularly for highly specialized care and precision medicine (Pronovost, 2013). Teaming up across professions, specialties, and across the authority gradient does not come naturally (Edmondson, 2012). Healthcare providers, universities, and training institutions include teamwork and the ability to collaborate in any healthcare team in their learning objectives. In particular, simulation-based teamwork training allows both students and professionals to practice and reflect on teamwork skills in meaningful settings without putting patients at risk (Weaver et al., 2014; Hughes et al., 2016). To be effective, training should be guided, and teamwork performance should be measured (Salas et al., 2009). Without measuring teamwork, feedback and debriefing conversations—and ultimately learning—will be limited (Rosen et al., 2008; Rudolph et al., 2008; Fey et al., 2022). However, identifying relevant teamwork behaviors and tracking them in complex, dynamic, and fast-paced simulated clinical situations is challenging (Halgas et al., 2022). Observing and measuring teamwork in action is prone to bias and constitutes a significant cognitive load even for trained instructors (Caverni et al., 1990; Greig et al., 2014; Uher and Visalberghi, 2016; Fraser et al., 2018). Additionally, simulation educators vary in individual expertise, and feedback might differ between them (Shrivastava et al., 2010; Bosse et al., 2015). We aim to support educators by contributing to the sophisticated collection of dynamic teamwork data (Petrosoniak et al., 2019; Marcelino et al., 2020; Shuffler et al., 2020; Wiltshire et al., 2020; Abegglen et al., 2022).

The choice of how to measure teamwork impacts the possibilities of further data use. For example, while using behavioral anchored rating scales (BARS) is relatively easy, it rarely provides enough variance in the acquired data and only limited information on temporal matters (Kolbe et al., 2013; Dietz et al., 2014; Brauner et al., 2018). On the other hand, timed, event-based behavior coding of teamwork behavior provides more information on the time and duration of behaviors but is complex and time-consuming (Brauner et al., 2018). Although event-based behavior coding allows for reliably capturing many explicit and verbal teamwork behavior (e.g., giving instructions or providing information on request) and allows for capturing more implicit teamwork behavior as well (e.g., team member monitoring), it usually suffers low interrater reliability (Kolbe et al., 2013; Uher and Visalberghi, 2016; Brauner et al., 2018). Low-quality data on team performance impair correct conclusions about team processes and performance, enhance the risk of negative learning, and limit training capacities (Salas et al., 2009).

We propose that using technology to objectively, continuously, and reliably measure teamwork dynamics will improve the quality of teamwork performance data in simulation-based training in healthcare. Technology-based measurement is a promising and fast-developing field of team science that can offer many opportunities for quantitative, scalable, objective, repeatable, new ways of recording data and resulting feedback conversations based on video data (Kozlowski, 2015; Klonek et al., 2019; Kolbe and Boos, 2019; Halgas et al., 2022). Teamwork metrics derived from technology can measure multiple behaviors simultaneously and allow for continuous observation of all team members over the duration of the simulation. They could be especially helpful for observing more implicit behaviors and team interactions that are not detectable via observation by humans (Uher and Visalberghi, 2016). Once established, technology-based metrics are reproducible and could be used for measuring teamwork dynamics during training and research.

Sensor-based measurement and wearable technologies have the ability to capture team dynamics (Rosen et al., 2014; Halgas et al., 2022). For example, Radio-Frequency Identification Devices (RFID) have been successfully used to measure the proximity between team members (Isella et al., 2011) and distance traveled during nursing shifts (Hendrich et al., 2008). In another study heart rate sensors allowed assumptions regarding the physiological synchronization of surgical teams (Dias et al., 2019). In this observational study, we explored the use of video-based technologies for continuously measuring teamwork behavior during simulation-based training in healthcare. We investigated two minimally invasive, video-based technologies: eye tracking and pose estimation.

**Figure 1:**
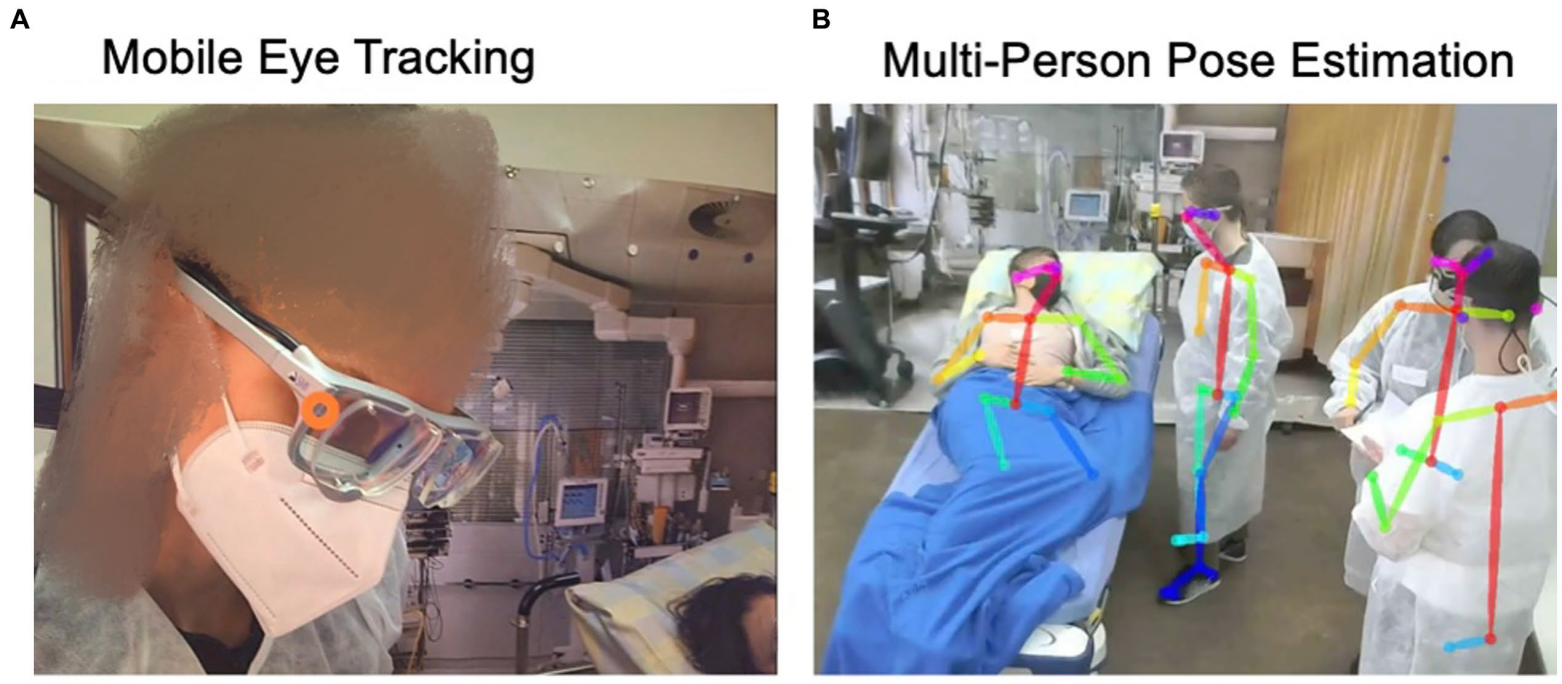
Illustration of mobile eye tracking **(A)** and multi-person pose estimation **(B)**.

Mobile eye tracking, an established wearable and minimally invasive technology in the field of healthcare devices and training (Henneman et al., 2017; Weiss et al., 2021), measures what a team member wearing the glasses is looking at (Figure 1A). We used eye tracking and its resulting data to precisely calculate the occurrence and length of eye contact between team members. Eye contact occurs naturally in conversation and is especially relevant during listening communication patterns (Ruth, 1992; Bohannon et al., 2013). Therefore, we considered eye contact a valuable metric for teamwork (Vertegaal et al., 2001; Fasold et al., 2021).

The second video-based technology we investigated was multi-person pose estimation as newer, non-wearable technology (Cao et al., 2021; Weiss et al., 2023). It measures human pose by calculating the exact position of human joints (Figure 1B). Combining two simultaneously recorded video data sets of each team allows for calculating the 3D position of all team members and, thus, their positioning to each other. We calculated each individual’s distance to the patient and the team members. The distance to patient influences the healthcare providers’ relationship with them (Schnittker, 2004) and is relevant during the workflows of teams (Petrosoniak et al., 2019) and movement coordination (Alderisio et al., 2017). Distance and movement may allow educators to make assumptions regarding the quality of team coordination (Petrosoniak et al., 2019; Marcelino et al., 2020; Shuffler et al., 2020; Tolg and Lorenz, 2020; Wiltshire et al., 2020), therefore being a relevant measure for teamwork. In summary, the ability to precisely measure and visualize eye contact and team member pose over time is highly relevant for simulation-based training providers. It allows an automated and dynamic capturing of visual attention, eye contact, team member positioning, and distance. Being aware of our own and team’s attention and positioning enables learning.

This study aims to explore the use of mobile eye tracking and multi-person pose estimation to continuously collect data and measure teamwork during simulation-based training in healthcare. This is an essential step that will enable further studies validating eye tracking and multi-person pose estimation metrics. These technology-based metrics intend to complement existing methods of teamwork assessment, support simulation faculty, improve the quality of simulation-based training and build examples for new methods of measuring teamwork based on technology.

## 2. Methods

### 2.1. Study design

**Figure 2:**
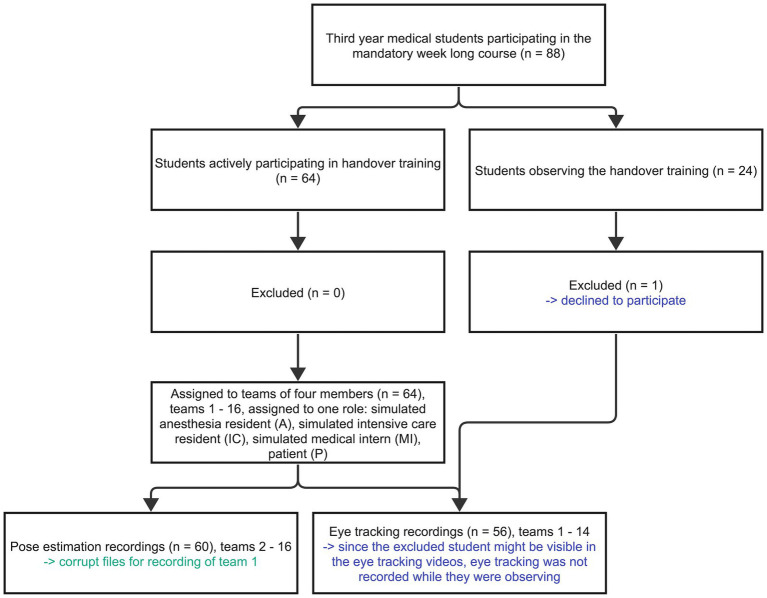
Participant’s flow diagram visualizes the participants, including their enrollment, consent, distribution, and data sets. The excluded participant is highlighted blue and the corrupt file green.

We conducted this observational study during a week-long, simulation-based training in March 2022 with a convenience sample. Third-year medical students of ETH Zurich (Zurich, Switzerland) participated in this training conducted at the simulation center of the University Hospital Zurich (Zurich, Switzerland). The training included eight four-hour simulation exercises on clinical teamwork situations. The overall 88 eligible students rotated in teams of 10–12 students through the course. We conducted this study in one of the eight clinical teamwork simulations, with the topic patient handover. The inclusion criteria were third-year medical students, trackable eyes, and participants’ consent. Of the eligible 88 students, 64 actively participated in the simulation scenarios, while the remaining 24 students observed the scenarios and participated in the subsequent debriefings (Figure 2).

### 2.2. Study ethics

This study was granted exemption from the ethics committee of canton Zurich, Switzerland (BASEC number: Req-2020-00200). No patients were involved, study participation was voluntary, and participants’ written informed consent was obtained.

### 2.3. Simulation-based training and handover case

We used a handover simulation scenario for data collection to explore the applicability of eye tracking and multi-person pose estimation. During patient handover, healthcare providers communicate information and responsibility about patients to ensure their continued, safe care during transfers among units or shift changes (Foster and Manser, 2012). Teamwork is critical during handover (Bogenstätter et al., 2009; Desmedt et al., 2021). The training’s learning objectives included the ability to describe pitfalls and risk management strategies such as iSBAR, a communication rubric to standardize team communication during handover (Müller et al., 2018). Two formally trained, experienced simulation educators with a nursing background in intensive care led the handover training. They introduced students to the course, aimed to establish and maintain a psychologically safe learning space, allowed students to familiarize themselves with the particular setting, and oriented them toward the learning objectives (Rudolph et al., 2014; Kolbe et al., 2020). After the introduction, a member of the study team and two master students explained the study goals and recording technologies, invited students to participate, and asked for informed, written consent.

The simulated case included a patient who had undergone trauma surgery after a bicycle accident to be handed over from surgery to the intensive care unit. A room in the simulation center was prepared with a bed and pictures of intensive care unit (ICU) settings. One member of the student team presumed the role of the patient (P) lying in bed. The other three students assumed the roles of anesthesia resident physician (A), intensive care resident (IC), and medical intern (MI). The scenario started with A & IC distancing themselves from P while MI took care of P. The patient was instructed to feel nauseous and in pain, challenging the team members to continue a structured handover. Team members had to take care of the patient while engaging in a structured handover. After the scenario, the two simulation instructors led debriefings based on the Debriefing with Good Judgment approach (Rudolph et al., 2007), which lasted approximately 45 min.

### 2.4. Data recording

**Figure 3:**
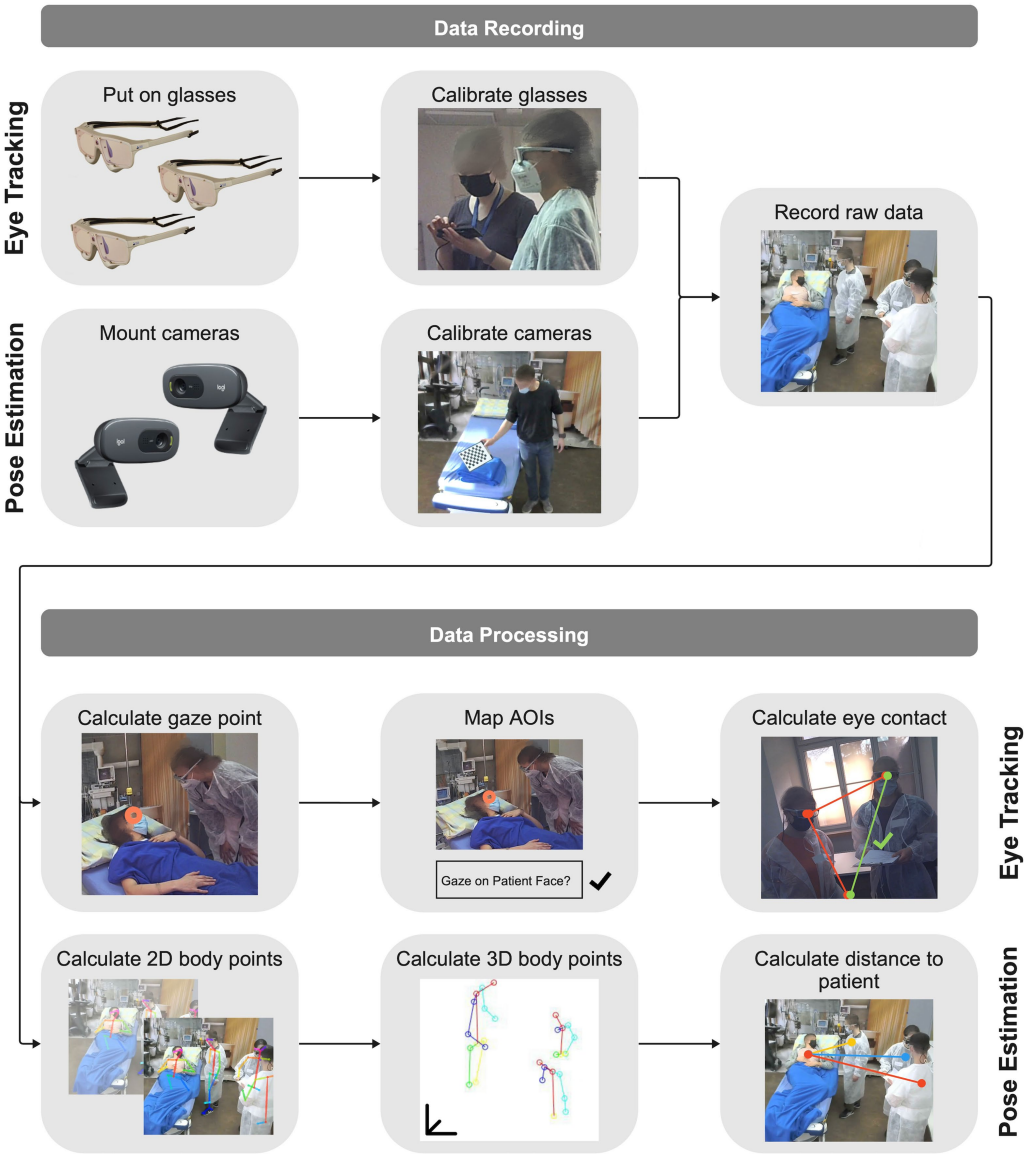
Process of data recording with measuring technologies to raw data and data processing from raw data to teamwork metrics for mobile eye tracking and multi-person 3D pose estimation.

For mobile eye tracking, we used three SMI ETG 2 Wireless mobile eye tracking glasses (Figure 3, Senso Motoric Instruments, Teltow, Germany). We calibrated the eye tracking glasses for every participant with a three-point calibration technique. The glasses recorded the eyes of the participants and their point of view, including audio, therefore allowing us to calculate the gaze point. After each use, we disinfected the glasses.

For multi-person pose estimation, we used two Logitech C270 webcams (Logitech, Lausanne, Switzerland) to record videos of the simulated cases (Figure 3). The cameras were neither invasive nor wearable, therefore not limiting the immersion of the participants or taking time to mount on their bodies. We mounted two pose estimation cameras on the ceiling and calibrated them once using a checkerboard.

### 2.5. Data processing

We used SMI BeGaze 3.6 (Senso Motoric Instruments, Teltow, Germany) to process the mobile eye tracking data (Figure 3). This software calculated the gaze point, the data of in what millisecond which person is focusing on, in each individual frame. Afterward, we defined the areas of interest (AOIs), relevant and visible objects, people, backgrounds we want to base our analysis on. The AOIs were: face MI, body (excluding the face) MI, face A, body A, face IC, body IC, face P, body P, room, and patient sheet. We manually mapped the gaze point to the AOIs for each frame, for example if the gaze point focused on the patient face we mapped it to the face P AOI. Finally, we exported the mapped AOI data and further processed it using MATLAB (MathWorks, Natick, Massachusetts, USA): we calculated the eye contact time between the team members and visualized using the face AOIs. Additionally, we visualized the complete visual attention of the team members by plotting on which AOI each team member was focusing over time.

We used the open-source software OpenPose (Cao et al., 2021) to process the recorded pose estimation videos (Figure 3). OpenPose allows for detecting 2D human body skeleton points, e.g., chest, shoulder, and hand, for all team members. No body markers were needed, which makes this method completely non-invasive and, despite its limitations, the accuracy of this methodology is sufficiently high to warrant its use. We exported the resulting two 2D pose estimation data sets — one for each camera — and used MATLAB (MathWorks, Natick, Massachusetts, USA) for triangulation, resulting in 3D human body skeletal points. With the 3D representation of the team members and the patient, using their chest points, we calculated the distances between each team member to the patient, as well as the distance between the team members for every frame. Subsequently, we obtained and visualized the average distances. For both technologies, we calculated the statistics (Kruskal-Wallis tests) using SPSS Statistics 28 (IBM, Armonk, New York, USA).

## 3. Results

### 3.1. Recorded data and participants

Sixty four students organized in 16 teams of four assumed an active role during the simulated cases of this study. The simulated roles were anesthesia resident physician (A), intensive care resident (IC), medical intern (MI), and patient (P). The student’s demographics are shown in Table 1. Since one student observing teams 15 and 16 chose not to participate in the study, no eye tracking was recorded since the particular student might have been visible. Therefore, we collected eye tracking data from 14 teams (teams 1–14), with three mobile eye tracking glasses per team resulting in 42 eye tracking data sets. Since the pose estimation cameras were fixed and only recorded the team itself, we did not have to exclude the videos of teams 15 and 16. Thus, we recorded all 16 teams with pose estimation. The data set of team 1 was not usable, resulting in overall usable 15 pose estimation team data sets, which allowed us to calculate 60 individual human pose estimation data sets (see Figure 2). The average simulation case length was 6.72 min [min 4.08 min – max 9.57 min], with a combined length of all cases resulting in 107.57 min.

**Table 1:** Participant characteristics (*n* = 64), including average age in years (± SD), the female and male sex ratio in percent, and the percent of participants having completed their obligatory nursing internship.

Participant characteristics	
Age (years)	22.45 ± 1.85
Female sex (%)	57.81
Male sex (%)	42.19
Nursing internship completed (%)	65.63

### 3.2. Eye tracking—eye contact

**Figure 4:**
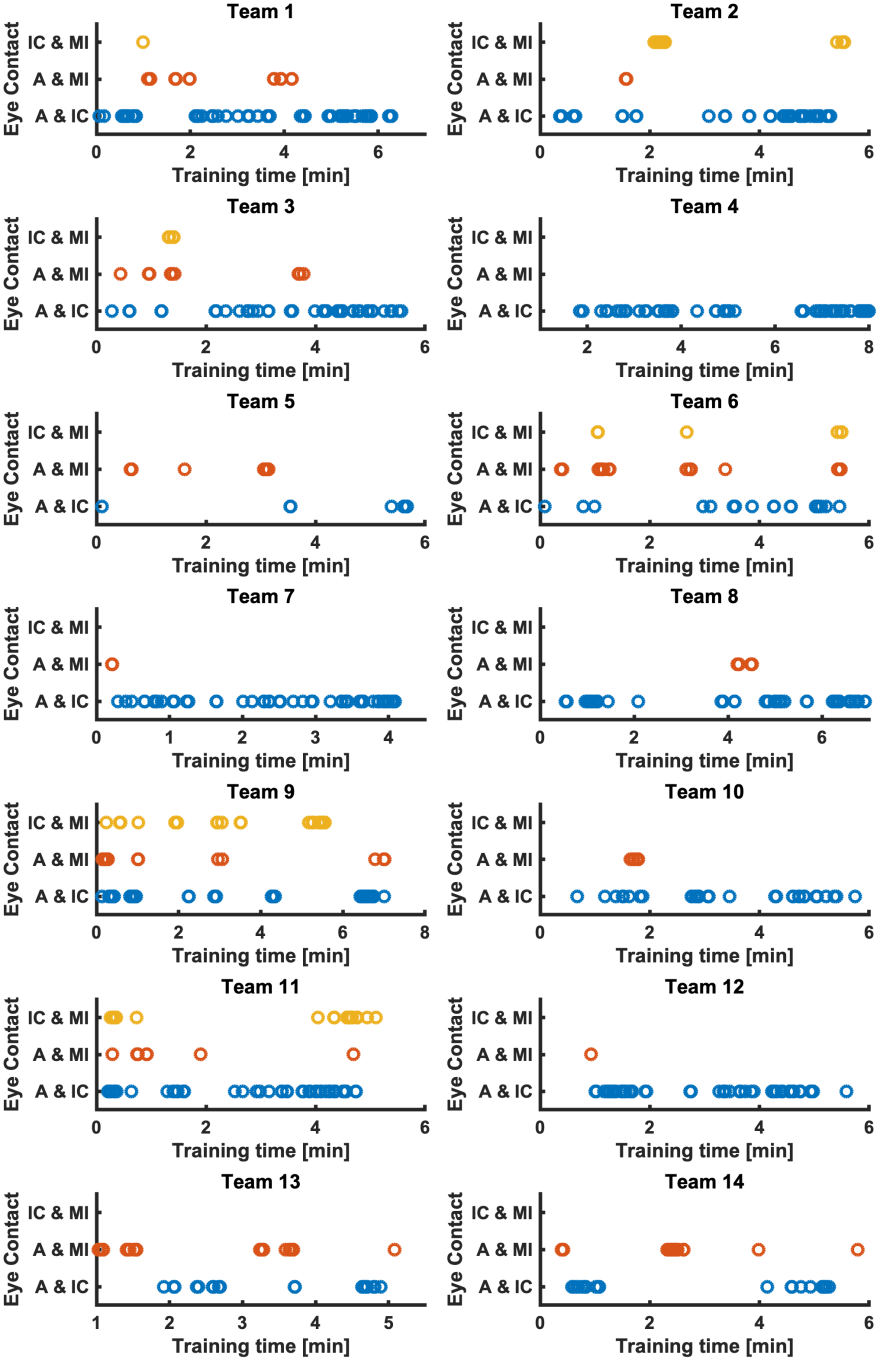
Eye contact between A & IC (blue), A & MI (red), and IC & MI (yellow) of all teams over training time in minutes. A, Simulated Anesthesia Resident; IC, Simulated Intensive Care Resident; MI, Simulated Medical Intern.

The measured eye contact times, i.e., when team members looked each other in the eye, for all teams and their members are visible in Table 2; Figure 4. The average eye contact times for all teams were 14 s for A & IC, 3.38 s for A & MI, and 1.99 s for IC & MI (H(2) = 19.029, *p* < 0.001) with an average eye contact time of 6.46 s for all teams and roles. Eye contact times varied extensively between teams.

**Table 2:** Eye contact in seconds for all teams, including the average of all teams, between the different team members depending on their roles (A, Simulated Anesthesia Resident; IC, Simulated Intensive Care Resident; MI, Simulated Medical Intern), high eye contact times (over 20 s) are highlighted bold while low eye contact times (below 2 s) are highlighted *cursive*.

Eye contact between team members [s]			
Team number	Team member roles		
	A & IC	A & MI	IC & MI
Team 1	**27.94**	4.88	*0.02*
Team 2	16.21	*1.15*	5.47
Team 3	11.38	2.71	*1.44*
Team 4	**28.01**	*0.00*	*0.00*
Team 5	2.36	3.93	*0.00*
Team 6	4.82	7.24	*1.29*
Team 7	13.45	*0.23*	*0.00*
Team 8	14.09	*1.89*	*0.00*
Team 9	**25.50**	7.39	12.74
Team 10	9.62	*1.75*	*0.00*
Team 11	11.29	*1.87*	6.97
Team 12	**20.26**	*0.38*	*0.00*
Team 13	5.23	5.29	*0.00*
Team 14	5.92	8.69	*0.00*
Average of all teams	14.00	3.38	*1.99*

**Figure 5:**
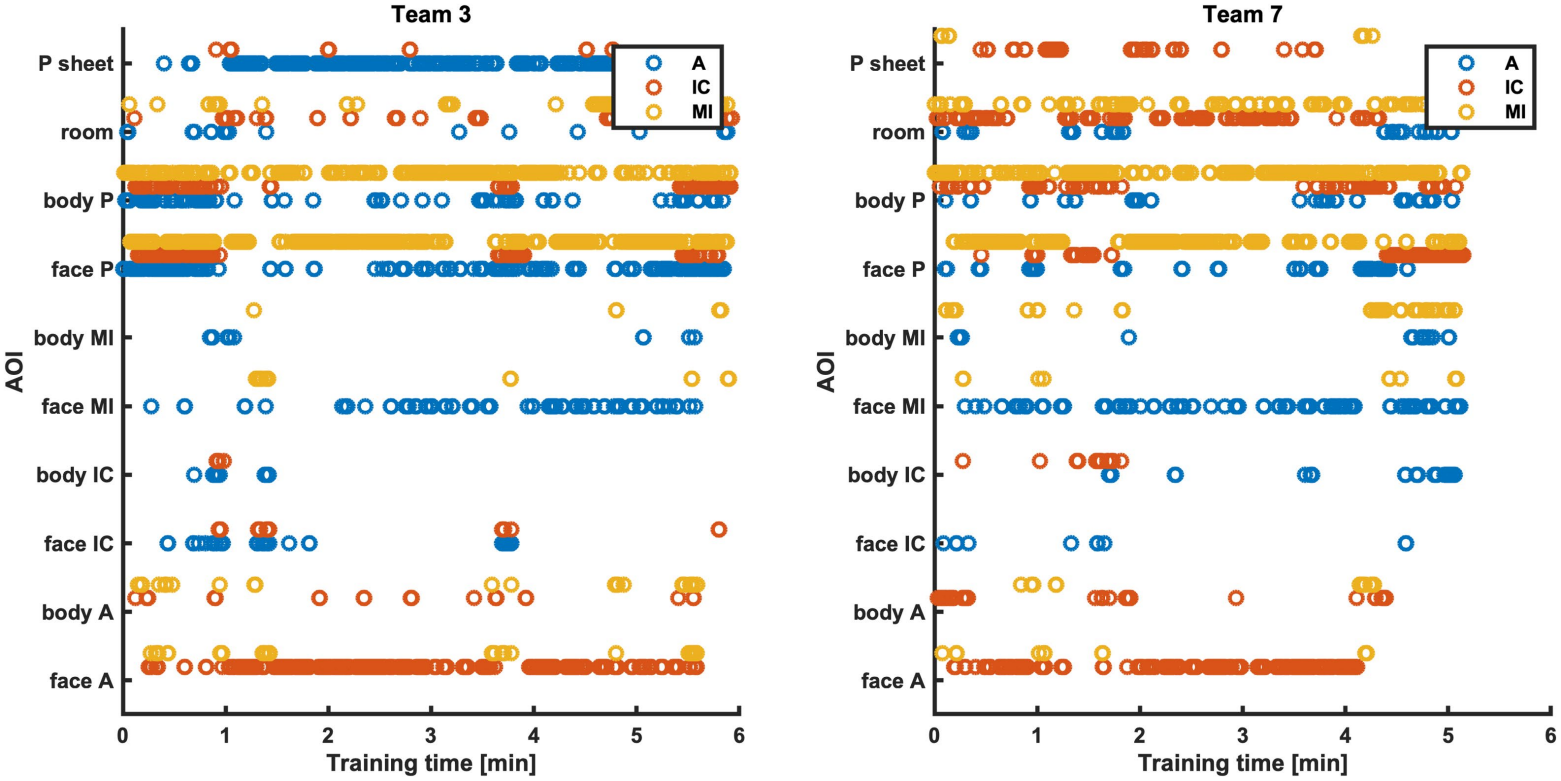
AOIs the team members (A blue, IC red, MI yellow) focused on during the training time, for example teams 3 and 7. A, Simulated Anesthesia Resident; IC, Simulated Intensive Care Resident; MI, Simulated Medical Intern.

An additional measure based on the eye tracking data, is the visualization of all team member’s gaze points over the whole time of the simulation. On which AOI each team member focuses on during the simulation is visualized for two example teams in Figure 5.

### 3.3. Multi-person pose estimation—distance to patient

**Figure 6:**
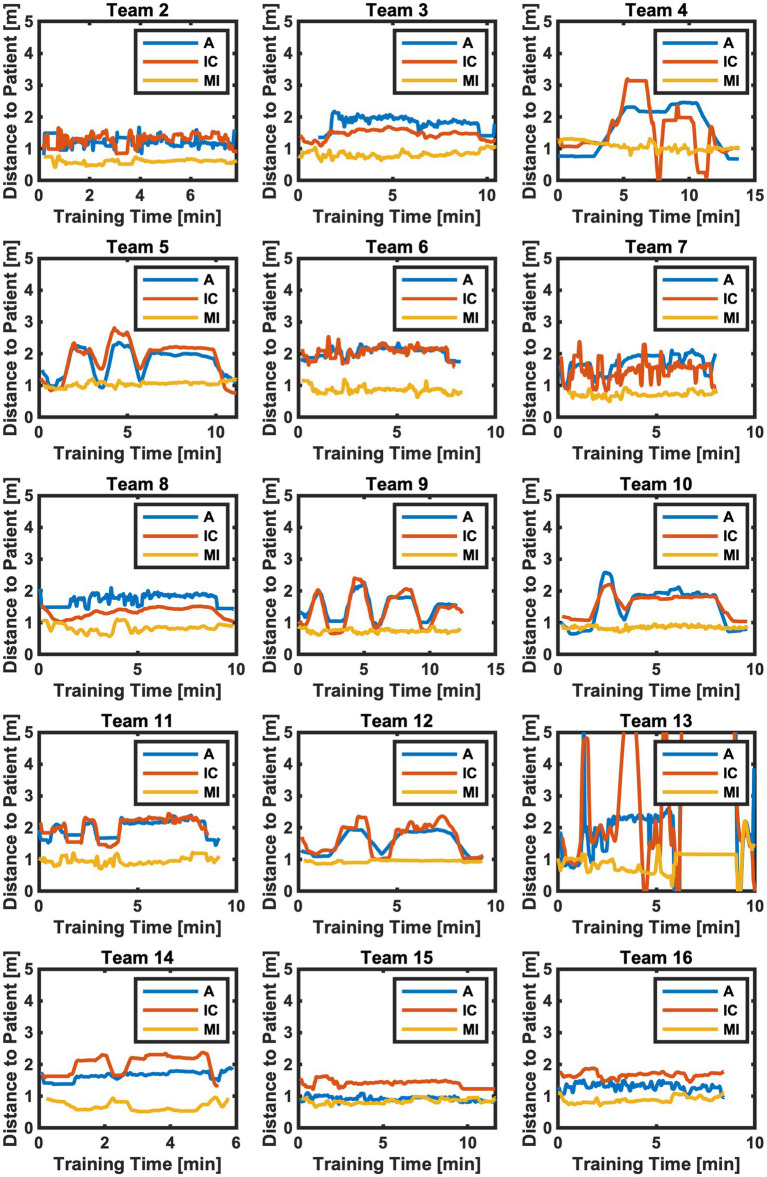
Distance to patient for all teams and team members (A blue, IC red, MI yellow) over the training time. A, Simulated Anesthesia Resident; IC, Simulated Intensive Care Resident; MI, Simulated Medical Intern.

**Figure 7:**
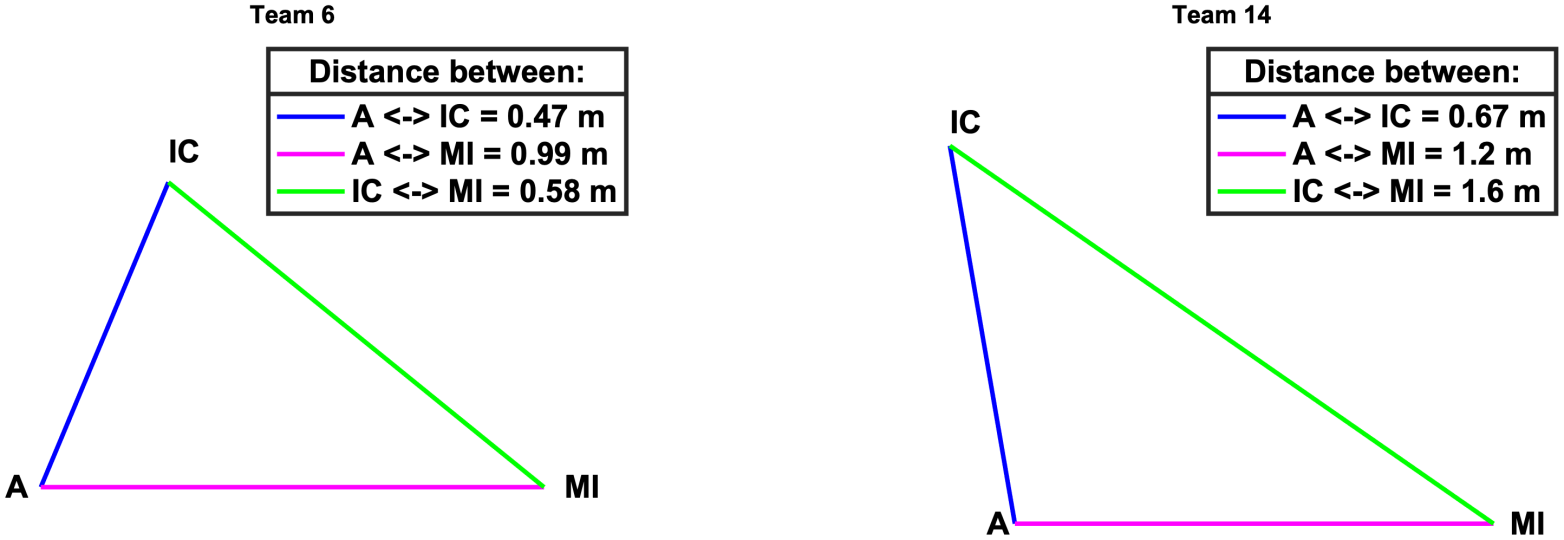
Average distance between team members (A & IC blue, A & MI magenta, IC & MI green) for example teams 6 and 14. A, Simulated Anesthesia Resident; IC, Simulated Intensive Care Resident; MI, Simulated Medical Intern.

The calculated distance to the patient from team members A, IC, and MI is visualized in Figure 6 over the time of the simulated case. The average values for each team are presented in Table 3. The average distance over all teams results in 1.15 m for A, 1.11 m for IC, and 0.78 m for MI (H(2) = 16.642, value of *p* <0.001), with an average distance of 1.01 m to the patient for all teams and roles. The average distance between team members based on calculated 3D pose estimation data is visualized for two teams as an example in Figure 7.

**Table 3:** Distance to patient in meters for all teams and team members, including the average of all teams (A, Simulated Anesthesia Resident; IC, Simulated Intensive Care Resident; MI, Simulated Medical Intern), high distances to patient (over 1.5 m) are highlighted bold while low distances (below 0.5 m) are highlighted *cursive*.

Distance to Patient [m]			
Team number	Team member		
	A	IC	MI
Team 2	*0.44*	0.94	*0.43*
Team 3	**1.53**	1.35	0.72
Team 4	1.04	0.52	1.07
Team 5	**1.53**	1.15	1.01
Team 6	**1.56**	**1.56**	0.78
Team 7	1.05	0.65	0.71
Team 8	1.16	1.23	0.78
Team 9	1.16	1.14	0.75
Team 10	0.68	1.34	0.86
Team 11	1.28	1.31	0.89
Team 12	**1.53**	0.65	0.93
Team 13	0.84	*0.32*	*0.42*
Team 14	**1.53**	**1.55**	0.70
Team 15	0.84	1.32	0.81
Team 16	1.11	**1.60**	0.85
Average all teams	1.15	1.11	0.78

## 4. Discussion

This study explored the use of video-based, minimally invasive technologies to collect data to measure teamwork in simulation-based training in healthcare. We found that both technologies reliably recorded and analyzed data, only one pose estimation data set was unusable. In what follows, we discuss the feasibility, contribution, and limitations of this study.

### 4.1. Feasibility of data collection and processing

Mobile eye tracking allowed for precise measurement of visual attention while being minimally invasive. Some participants reported casually and by themselves that they had forgotten that they were wearing the glasses while removing the mobile eye tracking glasses. However, completely non-invasive eye tracking would be beneficial. Although remote eye tracking is common, it currently cannot be used for moving participants (Ferhat and Vilariño, 2016). Handling the mobile eye tracking glasses was time-intensive during the recording since the glasses needed to be calibrated for every team member. However, the collected data yielded valuable, complex details on teamwork. We were able to track three team members simultaneously while not losing a single data set. During data processing, we had to manually map the AOIs, which was time-consuming. Automation of this processing step is being developed (Wolf et al., 2018).

The recording of multi-person pose estimation was more effortless. The one-time calibration for all recordings took little time. The method was entirely non-invasive, neither distracting participants nor hindering their immersion in the simulation. Unfortunately, one data set was unusable. We assume that the video files were corrupted during the process of being saved to the hard drive. During data processing, having multiple participants in the same camera frame was challenging. If occlusions occurred, no data could be extracted about a person if their body was not visible. We manually checked the indexes of the team members to ensure that the algorithm did not mix up the team members. A promising solution to this problem might be using depth cameras or more webcams to record data from multiple points of view.

### 4.2. Contribution of results for measuring teamwork in healthcare

The teamwork metrics that were calculated and visualized in this paper show the applicability of eye tracking and pose estimation to measure teamwork. Both mobile eye tracking and multi-person pose estimation allowed for collecting numerous, continuous data. The challenge—as with any technology-driven teamwork measure—is to identify parameters that matter and serve to discriminate among teams (Klonek et al., 2019). In our view, using both eye tracking and pose estimation allowed not only for precisely measuring and visualizing eye contact (Figure 4) and distance among patient and team members (Figure 6). It also allowed for discrimination between teams: eye contact among team members and distance to patient (and among team members) varied extensively from team to team. For example, all members of Team 9 had eye contact among each other numerous times (Figure 4). In contrast, members of Team 5, only A made eye contact with MI and IC a few times, while MI and IC had no eye contact at all. In Team 4, A and IC had exclusively much eye contact among each other, while A and MI and IC and MI did not look at each other (Figure 4). The visualization of every team member’s visual attention during the whole scenario duration (Figure 5) might be very interesting to investigate teamwork.

Regarding distance to patient, all members of Team 15 and 16 had little distance to the patient and slight variance in the distance over time (Figure 6). In contrast, members of Team 13 heavily varied their distance to the patient among each other and over time (Figure 6). That is, both metrics indicate sensitivity to differences in team processes. Neither eye contact nor pose tracking are possible with the naked eye. Yet, for teamwork in healthcare, certain interaction patterns may make all the difference for patient care (Kolbe et al., 2014; Su et al., 2017; Schmutz et al., 2019). The ability to precisely measure and visualize eye contact and team member pose over time is highly relevant for simulation-based training providers: it allows for more automated and dynamic capturing of visual attention, eye contact, team member positioning, and distance. Simulation educators can access this data and use it for discussing matches and mismatches in desired team performance during debriefing. For example, visual attention and eye contact data can serve discussions of situation awareness, power, and speaking up (Dovidio and Ellyson, 1982). Distance measures may provide essential details in discussing team coordination and task management. For example, in our simulated handover case, A and IC were instructed to distance themselves from P and MI to discuss the patient information, while MI should stay close to P to take care of them. If metrics depending on the teams’ position and movement are developed and validated, pose estimation allows continuous measuring of them, allowing for testing hypotheses and performance matches.

These technology-based metrics may complement behavior observation without replacing traditional methods. Medical competence assessment, especially of teamwork, needs both analytic and holistic approaches (Rotthoff et al., 2021), and mobile eye tracking and multi-person pose estimation allow to draw analytical conclusions in a more complex setting than before. An example of combining multiple methods could include self-reports of participants, supporting reflective practice (Liaw et al., 2012), technology-based metrics providing analytical observations for specific behaviors, and expert assessors observing the general behavior based on their extensive knowledge. The vision of using technology to measure teamwork is a static and fully automated recording set-up based in a simulation center. With this set-up new teamwork metrics can be easily co-created and validated with experts and subsequently used to support training. When experts find a new competence metric based on visual attention or body position, we can analyze it with our recorded data set if the participant’s consent allows it. The practical applications today are to provide educators with visualizations of existing metrics after the simulated case to use during debriefing. For example, learners may watch their parts of the recorded simulation, including the metrics, which may increase learning (Farooq et al., 2017; Gordon et al., 2017). Recording expert teams performing challenging teamwork tasks may be used in teamwork training to set masterly learning goals and provide specific guidance during rapid cycle deliberate practice (Barsuk et al., 2016; Salvetti et al., 2019; Ng et al., 2021). Our study focuses on teams of three simulated healthcare professionals and one patient to not rely solely on research with dyads to conclude the use of wearable technology in team contexts (Kazi et al., 2021; Halgas et al., 2022). Although the metrics are developed and visualized for a handover scenario, they can easily be transferred to other training scenarios.

### 4.3. Limitations and further research needs

Our study has limitations. First, although eye tracking and multi-person pose estimation showed promising opportunities and relevance, they require more validation research. In particular, indicators for criterion validity were not included in our study and are highly needed. That is, we cannot conclude if teams with a certain degree of eye contact or distance to patient performed better or worse. This is important, though, and should be studied with experienced healthcare teams rather than with a student sample.

Second, although the AOIs provided a rich set of dynamic details, their information density is high: they provided details about what each team member is looking at and how that changes over time (Figure 5). This level of detail and complexity might be too overwhelming to support simulation educators during debriefings. Simpler indices and/or visualizations will be needed to enhance the applicability of results. However, researchers might find it interesting to discover teamwork patterns in visual behavior. For example, seeing patients enhances the learning (Larsen et al., 2013), which can be measured by focusing on the two patient-related AOIs.

Third, the pose estimation teamwork metric of the team’s distance to the patient and between the team members may have been influenced by the COVID-19 situation. During data collection in March 2022 people were required to observe the social distance (Jarvis et al., 2020).

Fourth, the process of calculating the first metric for both measures was complicated and time intensive. Fortunately, every iteration and further metric was faster because the data processing framework was already established. Therefore, processing newly recorded data using existing metrics or developing new metrics and analyzing existing data takes lower effort and is faster. Additionally, the recorded and calculated data sets can be analyzed using other methods even years later, such as behavior coding or emerging machine learning techniques.

Fifth, we only studied one particular simulated case; the resulting metrics reflect only the interaction during simulated handover. Sixth, future studies may include the investigation of simulation educators’ cognitive load and overall training quality when using technology-based teamwork metrics (Fraser et al., 2018). Furthermore, the degree of acceptance of the methodologies by the participants may be quantified in future studies.

Finally, conducting this study required an interdisciplinary research team consisting of mechanical engineers and a team of healthcare simulation scientists. Currently, for using technology-based metrics to measure teamwork, interdisciplinary skills are essential: Technical knowledge is needed to program metrics and automate the process, while healthcare and teamwork knowledge is required to define relevant behaviors and metrics. However, once the technology is set up for data collection and the metrics are implemented, they will reduce the cognitive load of researchers and educators because complex team dynamics can be feasibly assessed during simulation-based teamwork training.

## 5. Conclusion

In this study, two minimally invasive video-based technologies, mobile eye tracking and multi-person pose estimation, were integrated into simulation-based healthcare training to measure teamwork. Both allowed the recording of objective, continuous, and reliable data that could be processed to multiple teamwork metrics. Future research in necessary to generalize our findings and how they may complement existing methods, support instructors, and contribute to the quality of teamwork training in healthcare.

## Data availability statement

The raw data supporting the conclusions of this article will be made available by the authors, without undue reservation.

## Ethics statement

The studies involving human participants were reviewed and approved by Ethics Committee of Canton Zurich, Switzerland. The patients/participants provided their written informed consent to participate in this study.

## Author contributions

KW collected and analyzed the data and wrote the first draft of the manuscript. MK wrote sections of the manuscript. All authors contributed to the conception and design of the study, manuscript revision, read, and approved the submitted version.

## Funding

Open access funding by ETH Zurich.

## Conflict of interest

The authors declare that the research was conducted in the absence of any commercial or financial relationships that could be construed as a potential conflict of interest.

## Publisher’s note

All claims expressed in this article are solely those of the authors and do not necessarily represent those of their affiliated organizations, or those of the publisher, the editors and the reviewers. Any product that may be evaluated in this article, or claim that may be made by its manufacturer, is not guaranteed or endorsed by the publisher.

## Abbreviations

A: Simulated Anesthesia Resident
IC: Simulated Intensive Care Resident
ICU: Intensive Care Unit
MI: Simulated Medical Intern
P: Patient
AOI: Area of Interest
